# Repurposing simvastatin as a therapy for preterm labor: evidence from preclinical models

**DOI:** 10.1096/fj.201801104R

**Published:** 2018-10-12

**Authors:** Ashley K. Boyle, Sara F. Rinaldi, Adriano G. Rossi, Philippa T. K. Saunders, Jane E. Norman

**Affiliations:** * Tommy’s Centre for Maternal and Fetal Health, Medical Research Council (MRC) Centre for Reproductive Health, Queen’s Medical Research Institute, University of Edinburgh, Edinburgh, United Kingdom; and; † Medical Research Council (MRC) Centre for Inflammation Research, Queen’s Medical Research Institute, University of Edinburgh, Edinburgh, United Kingdom

**Keywords:** pregnancy, preterm birth, statins, inflammation, contraction

## Abstract

Preterm birth (PTB), the leading cause of neonatal morbidity and mortality, urgently requires novel therapeutic agents. Spontaneous PTB, resulting from preterm labor, is commonly caused by intrauterine infection/inflammation. Statins are well-established, cholesterol-lowering drugs that can reduce inflammation and inhibit vascular smooth muscle contraction. We show that simvastatin reduced the incidence of PTB in a validated intrauterine LPS-induced PTB mouse model, decreased uterine proinflammatory mRNA concentrations (IL-6, Cxcl1, and Ccl2), and reduced serum IL-6 concentration. In human myometrial cells, simvastatin reduced proinflammatory mediator mRNA and protein expression (IL-6 and IL-8) and increased anti-inflammatory cytokine mRNA expression (IL-10 and IL-13). Critically, simvastatin inhibited myometrial cell contraction, basally and during inflammation, and reduced phosphorylated myosin light chain concentration. Supplementation with mevalonate and geranylgeranyl pyrophosphate, but not farnesyl pyrophosphate, abolished these anticontractile effects, indicating that the Rho/Rho-associated protein kinase pathway is critically involved. Thus, simvastatin reduces PTB incidence in mice, inhibits myometrial contractions, and exhibits key anti-inflammatory effects, providing a rationale for investigation into the repurposing of statins to treat preterm labor in women.—Boyle, A. K., Rinaldi, S. F., Rossi, A. G., Saunders, P. T. K., Norman, J. E. Repurposing simvastatin as a therapy for preterm labor: evidence from preclinical models.

Spontaneous preterm labor (PTL) reportedly accounts for two thirds of cases of preterm birth (PTB). PTB is defined as delivery before 37 completed weeks of gestation and is responsible for 11.1% of annual births worldwide (1, 2). It is the leading cause of mortality in children aged <5 yr and has a substantial economic burden due to costs of neonatal intensive care and lifelong morbidity (3, 4). The etiology of PTL is poorly understood, but intrauterine infection/inflammation is believed to be the most common cause (5). Fetal exposure to this adverse intrauterine environment can result in fetal organ injury, particularly to the brain, increasing the risk of cognitive and neurologic impairment (6, 7).

Human labor is an inflammatory process associated with the influx of immune cells into the gestational tissues and the increased production of proinflammatory mediators, which stimulates uterine contractility, cervical ripening, and fetal membrane rupture (8, 9). Contraction of the myometrium is facilitated by the cross-bridging of the myofilaments actin and myosin, instigated by the increase of intracellular calcium (Ca2+) concentration and completed by the phosphorylation of myosin light chain (MLC) (10). Although drugs are often administered to reduce uterine contractility, there is little evidence to suggest that these tocolytic agents have either a substantial impact on the timing of delivery or improve the health of premature neonates (11, 12). New treatments are urgently needed that both suppress myometrial contractions and the inflammation associated with PTL, to both delay delivery and prevent fetal injury.

Statins are 3-hydroxy-3-methylglutaryl-coenzyme A reductase inhibitors. These widely used drugs are potent inhibitors of cholesterol biosynthesis, and they are commonly used for the prevention of cardiovascular disease. In addition, they are reported to have immunomodulation, anti-inflammatory, and antioxidative stress properties (13, 14). Of relevance to PTL, studies have also suggested that statins can exert anticontractile effects on vascular smooth muscle cells and endometriotic stromal cells, as well as on mouse and human myometrial tissue (15–19). These properties make statins potential candidates to repurpose for the prevention of PTB and the morbidity associated with it.

Further support for testing statins for PTB prevention is provided from animal and human studies that have described the use of statins for the prevention or treatment of pregnancy complications, such as intrauterine growth restriction, antiphospholipid syndrome, recurrent miscarriage, and preeclampsia (20–23). In addition, one study reported that statins could inhibit PTB by preventing cervical remodeling and myometrial contractions in a mouse model that induced early delivery by using intravaginal LPS (16). Notably, this study was small, and other groups have been unable to replicate PTB in this model.

In the current study, we investigated the effect of simvastatin treatment by using a well-validated intrauterine LPS-induced PTB mouse model (24) and complemented this method by measuring its impact on inflammation and contraction of human myometrial smooth muscle cells.

## MATERIALS AND METHODS

### Experimental compounds

Simvastatin (MilliporeSigma, Burlington, MA, USA) stock solution was prepared by dissolving 4 mg of the drug in 100 μl EtOH and 150 μl 0.1 N NaOH, followed by a 2-h incubation at 50°C. The stock solution was adjusted to pH 7 with HCl, and the volume was then made up to 1 ml using sterile PBS (Thermo Fisher Scientific, Waltham, MA, USA) and filter sterilized by using a 0.22 µm filter (MilliporeSigma). *Escherichia coli* LPS 0111:B4 (MilliporeSigma) was reconstituted in PBS, portioned into aliquots, and stored at −20°C. (±)-Mevalonolactone (MilliporeSigma) was diluted 1 in 10 in EtOH, then further diluted to 200 µM in PBS. Farnesyl pyrophosphate ammonium salt (F-PP; MilliporeSigma) and geranylgeranyl pyrophosphate ammonium salt (GG-PP; MilliporeSigma) were purchased in solutions of 2500 and 2000 µM, respectively. F-PP and GG-PP were diluted to 10 µM in PBS.

### Ultrasound-guided intrauterine LPS-induced PTB mouse model

All animal studies were conducted under a UK Home Office license to Jane E. Norman (70-8927) in line with the Animal Scientific Procedures Act (1986). Virgin female C57Bl/6 mice aged 6 wk were obtained from Charles River Laboratories (Margate, United Kingdom) and allowed to acclimate for 10 d before timed mating. Temperature (19–23°C) and humidity (∼55%) were tightly controlled at all times, with constant 12-h light/dark cycles. The ultrasound procedure was performed as previously described (24). Briefly, on gestational d 17 (d 1 designated when vaginal plug was found), mice were anesthetized by the inhalation of isoflurane (5% for induction, 1.5% for maintenance in oxygen) and positioned supine on the ultrasound stage. Abdominal hair was clipped and then removed with depilatory cream. Warm ultrasound gel was then applied to the abdomen. Scans were performed with the Vevo 770 High-Frequency Ultrasound Scanner (FujiFilm VisualSonics, Toronto, ON, Canada) with an RMV 707B probe (center frequency, 30 MHz). Temperature and heart rate were monitored throughout all procedures. Ultrasound was utilized to confirm the number of viable fetuses and then to guide injection of 1 µg LPS or PBS (25 μl) directly into the uterine lumen, between 2 gestational sacs, using a 33-gauge Hamilton syringe. Care was taken not to enter the amniotic cavity. Animals were allowed to recover from anesthesia and were monitored for signs of labor and delivery of pups. Cage cameras were used to monitor animals remotely. Time to delivery was recorded as the number of hours from the time of intrauterine injection of LPS/PBS to the delivery of the first pup. The number of live/dead pups was recorded within 24 h of their delivery. The percentage of live-born pups per litter was calculated by dividing the number of live pups found within 24 h of delivery by the number of viable pups counted *via* ultrasound on d 17.

### Simvastatin treatment of the PTB mouse model

Animals were randomly assigned to treatment groups, and the researcher was blinded to LPS/PBS treatment. On d 16, mice received an intraperitoneal injection of simvastatin (20 or 40 µg) or PBS. On d 17, 2 h after ultrasound-guided intrauterine injection, the mice received a second intraperitoneal injection of simvastatin or PBS. Experimental group numbers were determined according to a sample size calculation.

### Tissue collection

In a separate cohort of mice, uterus samples were harvested 6 h after intrauterine injection of LPS/PBS. Dams were euthanized by CO_2_ inhalation followed by exsanguination. Samples taken for quantitative RT-PCR (qRT-PCR) analysis were collected in RNAlater (MilliporeSigma), then stored at −80°C until required. Maternal blood was collected, allowed to clot, and then centrifuged at 2000 *g* for 20 min. The aliquoted serum was stored at −80°C. Amniotic fluid was collected from each gestational sac and pooled, resulting in 1 sample per dam, centrifuged at 8000 *g* for 10 min at 4°C, and then aliquoted and stored at −80°C.

### Human myometrial cells

Pregnant human myometrial 1–41 cells were isolated from a pregnant woman at term, immortalized, and selected by resistance to Geneticin (G418) (25). These myometrial cells (American Type Culture Collection, LGC Standards, Teddington, United Kingdom) were cultured as described elsewhere (26–28). Cells were routinely monitored for mycoplasma by using the MycoAlert Mycoplasma Detection Kit, according to the manufacturer’s instructions (Lonza, Slough, United Kingdom).

### Simvastatin treatment of myometrial cells

Cells were seeded at 1.5 × 10^5^ cells/ml in 6-well plates and partially serum-starved for 24 h at 37°C in (DMEM; Lonza) supplemented with 5% (v/v) charcoal-stripped fetal bovine serum (Thermo Fisher Scientific). Cells were stimulated with 100 ng/ml LPS and treated in duplicate either simultaneously with simvastatin (0.1, 10, 50 µM), pretreated with simvastatin, or posttreated with simvastatin and incubated for 24 h at 37°C.

### Cell metabolic activity

Cell metabolic activity was assessed for all treatments by measuring the ability of the cells to metabolize 3-(4,5-dimethylthiazol-2-*yl*)-2,5-diphenyltetrazolium bromide (MTT). Cells were seeded in triplicate at 10^4^ in 96-well plates and treated with simvastatin (0.1, 10, 25, 50 µM) alone or supplemented with LPS (25, 100 ng/ml), mevalonate (200 µM), GG-PP (10 µM), or F-PP (10 µM) for 24–48 h. MTT solution was applied for 4 h at 37°C. The medium and MTT solution were removed, and 100 µl acidified isopropanol was added for 20 min; light absorbance was then measured at 540 nm (Multiskan EX, LabSystems).

### Reverse transcription and qRT-PCR for human cell lysate and mouse tissue

Total RNA was extracted from cell lysate and mouse uterus samples by using an RNAeasy mini kit (Qiagen, Germantown, MD, USA) according to the manufacturer’s instructions, and quantified by using a NanoDrop 2000c (Thermo Fisher Scientific, Hemel Hempstead, United Kingdom). Total RNA (300 ng/µl) was reverse transcribed by using the High Capacity cDNA Reverse Transcription Kit (Thermo Fisher Scientific). Predesigned TaqMan gene expression assays (Thermo Fisher Scientific) were used to investigate genes of interest and are listed in **Table 1**. All qRT-PCR analyses were performed on a Thermo Fisher Scientific 7900HT instrument. Target mRNA expression was normalized for RNA loading using *β-actin* (Thermo Fisher Scientific), and the mRNA concentration in each sample was calculated relative to the vehicle control by using the 2^−ΔΔ*Ct*^ method of analysis.

**TABLE 1:** Predesigned TaqMan gene expression assay IDs

Gene	Species	Code
*IL-6*	Human	Hs00985639_m1
*IL-8*	Human	Hs00174103_m1
*IL-10*	Human	Hs00961622_m1
*IL-13*	Human	Hs00174379_m1
*Ccl2*	Mouse	Mm00441242_m1
*Cox-2*	Mouse	Mm00478374_m1
*Cxcl1*	Mouse	Mm04207460_m1
*Cxcl2*	Mouse	Mm00436450_m1
*Cx43*	Mouse	Mm01179639_s1
*Il-1β*	Mouse	Mm00434228_m1
*Il-6*	Mouse	Mm00446190_m1
*Il-10*	Mouse	Mm01288386_m1
*TNF-α*	Mouse	Mm00443258_m1

### Mouse and human ELISAs

Human myometrial cell supernatants were analyzed for IL-6 and IL-8 secretion by using DuoSet ELISAs (R&D Systems, Abingdon, United Kingdom). IL-6 was measured in mouse maternal serum and amniotic fluid (Quantikine; R&D Systems). Both assays were performed according to the manufacturer’s instructions.

### Collagen gel contraction assay

A collagen gel contraction assay was used to assess the effect of simvastatin on the contraction of myometrial cells and was performed as previously described (26–28). Briefly, myometrial cells (10^5^ cells/well) were embedded in type I rat tail collagen (Thermo Fisher Scientific). The collagen was allowed to polymerize overnight at 37°C. Treatments were prepared in 5% (v/v) fetal bovine serum DMEM, and wells were photographed at 0, 24, and 48 h by using a Leica MZ6 light microscope/camera (Leica Microsystems, Wetzlar, Germany). ImageJ software (National Institutes of Health, Bethesda, MD, USA) was used to analyze gel area. The gel area measurements at 24 and 48 h were calculated as a percentage of the mean gel area at the 0-h time point for each independent experiment.

### In-cell Western assay

In-cell Western analyses were performed to quantify phosphorylated myosin light chain (pMLC), as previously described (26, 27, 29). Briefly, myometrial cells were seeded at 2 × 10^4^ cells/well in black, 96-well, clear base plates (PerkinElmer, Waltham, MA, USA). The treatments were prepared in 5% (v/v) fetal bovine serum DMEM and applied in triplicate for 48 h at 37°C. Cells were fixed with 4% formaldehyde (MilliporeSigma) for 15 min at room temperature, and plates were then washed and permeabilized 3 times with 0.1% Triton X-100 in PBS for 5 min. The plates were incubated with primary antibodies, polyclonal rabbit anti-pMLC 2 [Ser19] (3671; Cell Signaling Technology, Danvers, MA, USA), and anti–α-Tubulin mAb (T9026; Sigma-Aldrich), and then secondary antibodies, 800CW and 680RD (926-68072 and 926-32213; Li-Cor, Lincoln, NE, USA). The Odyssey CLx Imaging System (Li-Cor) was used to read the plates and measure the signal in each well. The intensity of pMLC fluorescence was quantified relative to the α-Tubulin signal within the same well.

### Liquid chromatography tandem mass spectrometry quantification of serum progesterone

Progesterone concentration was measured in dam serum by liquid chromatography tandem mass spectrometry, using a QTrap 5500 (AB Sciex, Warrington, United Kingdom), with an Acquity Ultra Performance Liquid Chromatography (UPLC; Waters Corp., Manchester, United Kingdom). Mass spectral conditions are presented in **Table 2**. Analytes were extracted from serum (50 µl) supported liquid extraction (400 µl SLE+; Biotage, Uppsala, Sweden) with D9-progesterone (1.5 ng; C D N Isotopes, Pointe-Claire, QC, Canada) included as an internal standard. Separation took place at 40°C on an Ace Excel C18 (100 × 2.1 mm, 1.7 µm) column (Advanced Chromatography Technologies, Aberdeen, United Kingdom) using a gradient solvent system (50:50 of water with 0.1% formic acid and acetonitrile with 0.1% formic acid) with a gradient run of 6.5 min (**Table 3****)**.

**TABLE 2:** Mass spectral conditions for analysis of progesterone and the internal standard, D9-progesterone, utilizing positive and negative electrospray

Hormone	MW (Da)	Precursor ion (*m*/*z*)	Production quan; qual	Declustering potential (V)	Collision energy (V) quan; qual	Cell exit potential (V) quan; qual
Progesterone	314.462	315.0	97.0; 109.0	146	29; 37	10; 18
D9-Progesterone	323.52	324.1	100.0	151	31	38

Quan, quantifier ion; qual, qualifier ion.

**TABLE 3:** Chromatographic conditions (flow rate 0.5 ml/min)

Time (min)	Mobile phase A: water (0.1% formic acid, v/v)	Mobile phase B; acetonitrile (0.1% formic acid, v/v)
0	50	50
1	50	50
4	0	100
5	0	100
5.1	50	50
6.5	50	50

### Protein extraction and quantification

Tissue was homogenized in lysis buffer (RIPA buffer; MilliporeSigma) containing 1 Complete Protease Inhibitor Cocktail Tablet (Roche, Basel, Switzerland) using a Tissue Lyser II (Qiagen) at 25 Hz. The samples were incubated on ice for 5 min and then centrifuged at 10,000 *g* for 10 min at 4°C. The supernatant was then divided into aliquots and stored at −80°C. Protein was quantified by using the DC (Bio-Rad, Hercules, CA, USA) protein assay according to the manufacturer’s instructions.

### Western blot analyses

For connexin 43 (CX43) analysis, samples were loaded (20 µg protein/sample) on 4–12% (12-well) Bis-Tris precast NuPAGE gels and run in 3-(*N*-morpholino)propanesulfonic acid running buffer (Thermo Fisher Scientific) at 180 V for 70 min. The protein was transferred with the use of a wet-transfer system (100 V for 90 min) to Immobilon-FL PVDF membranes (MilliporeSigma). The membranes were blocked with 5% nonfat dry milk in 0.5% Tween (MilliporeSigma) Tris-buffered saline (Thermo Fisher Scientific) and incubated with primary antibodies, polyclonal rabbit anti-CX43 (ab11370; Abcam, Inc., Cambridge, United Kingdom), and monoclonal mouse anti–α-Tubulin overnight at 4°C. Membranes were incubated with secondary antibodies, 680RD and 800CW (Li-Cor), and scanned by using the Li-Cor Odyssey Fc Imaging System. The bands for CX43 and α-Tubulin were quantified by using the Odyssey analysis software.

Chemiluminescence was performed as previously described, with minor alterations. Briefly, the protein (45 µg protein/sample) was transferred to Immobilon-P PVDF membranes (MilliporeSigma) and blocked with 5% bovine serum albumin (MilliporeSigma) in 0.5% Tween Tris-buffered saline. After incubation with primary antibodies, monoclonal rabbit anti–IL-6 (12912; Cell Signaling Technology) and monoclonal mouse anti–α-Tubulin, and secondary antibodies, polyclonal swine anti-rabbit HRP (P0399; Agilent, Santa Clara, CA, USA) and polyclonal goat anti-mouse HRP (P0447; Agilent), the membranes were incubated with Amersham ECL Western blotting detection reagent (GE Healthcare Life Sciences, Marlborough, MA, USA) for up to 5 min, then scanned and analyzed as previously discussed.

### Statistics

Data are presented as means ± sem and were analyzed by using GraphPad Prism v.7 (GraphPad Software, La Jolla, CA, USA). For mouse studies, “*n*” represents the number of individual dams treated. For cell studies, “*n*” denotes the number of individual experiments performed, with the technical replicate number per experiment indicated in the figure legends. Time to delivery data were analyzed by using the Kruskal-Wallis test, with Dunn’s *post hoc* test. The percent data for live-born pups and collagen gel contraction were analyzed by performing an arcsine transformation on the proportions, to normalize the binomial distribution, followed by a 1-way ANOVA with either a Dunnett or Holm-Sidak *post hoc* test. ELISA and in-cell Western data were checked for normal distribution and square root transformed if necessary. These data, as well as qRT-PCR data, were then analyzed by using a 1-way ANOVA, followed by Dunnett’s *post hoc* test. Values of *P* < 0.05 were considered statistically significant.

### Study approval

All animal experiments were reviewed with the Named Training and Competency Officer and Named Veterinary Surgeon at the University of Edinburgh before commencing these studies.

## RESULTS

### Simvastatin treatment reduces the incidence of PTB in an LPS-induced mouse model

To initiate PTB, mice received intrauterine LPS *via* ultrasound guidance. As expected, and as we have previously reported (24), LPS administration induced significantly earlier delivery than when mice were treated with PBS (mean time to delivery, 29.7 ± 3.6 *vs.* 63.5 ± 2.3 h; *P* < 0.0001) (**Table 4** and Supplemental Fig. 1*A*). However, when mice were treated with 20 or 40 µg simvastatin in addition to LPS, the rate of PTB was reduced, and the mean time to delivery was significantly increased to 46.9 ± 6.5 and 45.3 ± 4.7 h, respectively (*P* = 0.0383 and 0.0469 *vs.* LPS).

**TABLE 4:** Time to delivery

Group	Time to delivery (h) *vs.* LPS
LPS	29.74 ± 3.6
Simvastatin (20 µg) + LPS	46.86 ± 6.5*
Simvastatin (40 µg) + LPS	45.3 ± 4.7*
PBS	63.54 ± 2.3****
Simvastatin (20 µg) + PBS	59.05 ± 2.2**
Simvastatin (40 µg) + PBS	52.84 ± 2.7

* *P* < 0.05, **P < 0.01, ****P < 0.0001.

Consistent with adverse effects of inflammation-induced PTB, a substantial reduction in the percentage of live-born pups was observed after LPS administration: a mean of 18.4% of pups per litter survived delivery compared with 87.7% in the PBS group (*P* < 0.0001) (**Table 5** and Supplemental Fig. 1*B*). In mice exposed to LPS, simvastatin treatment resulted in more live-born pups, but this difference did not reach statistical significance. As expected, mean serum progesterone concentrations paralleled time to delivery (Supplemental Fig. 2*A*).

**TABLE 5:** Percentage of live-born pups

Group	Live born pups (%) *vs.* LPS
LPS	18.43 ± 7.6
Simvastatin (20 µg) + LPS	40.39 ± 10.2
Simvastatin (40 µg) + LPS	39.18 ± 9.1
PBS	87.7 ± 4.7****
Simvastatin (20 µg) + PBS	88.29 ± 7.6****
Simvastatin (40 µg) + PBS	76.22 ± 7.6****

**** *P* < 0.0001.

### Simvastatin treatment reduces systemic inflammation as well as uterine inflammation and contraction-associated gene expression

**Figure 1:**
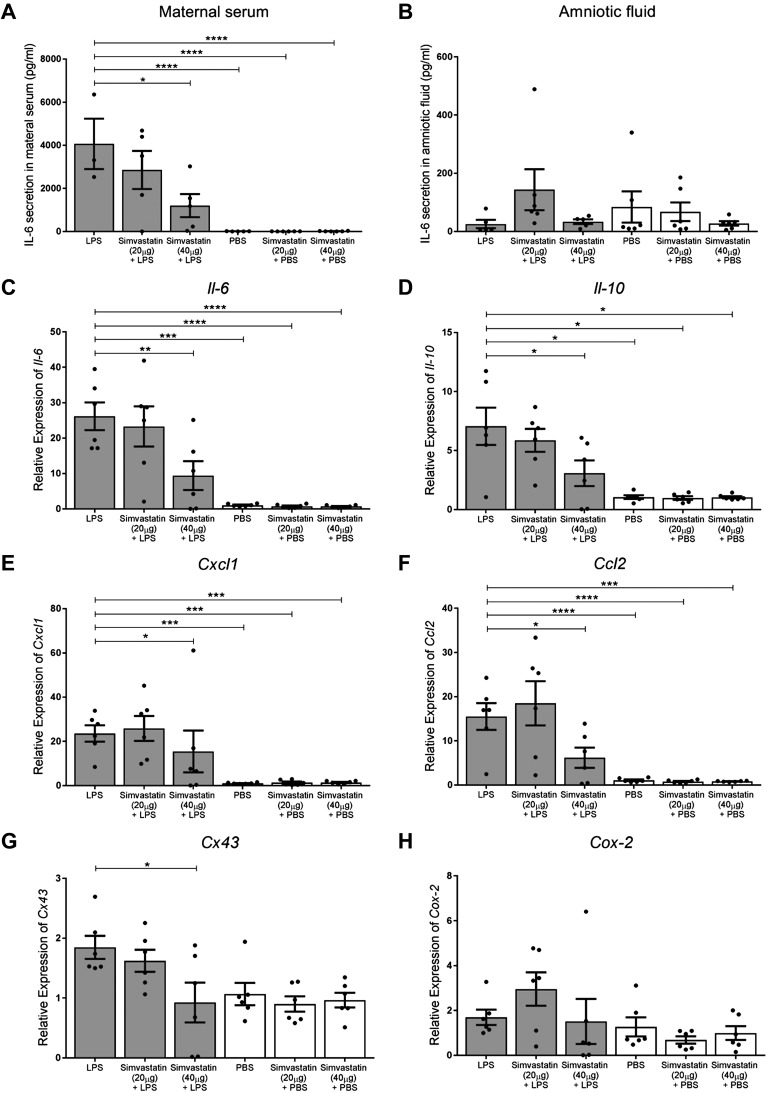
Simvastatin reduces circulating IL-6 concentration and down-regulates uterine inflammatory and contraction-associated mRNA expression. *A*) IL-6 concentration in the maternal serum was elevated 6 h after LPS treatment (*P* < 0.0001), which was attenuated by 40 µg simvastatin (*P* = 0.0213 *vs.* LPS); *n* = 3–6/group, 1-way ANOVA with Dunnett’s *post hoc* test. *B*) IL-6 concentration in the amniotic fluid was unaffected by LPS or simvastatin; *n* = 5–6/group. *C*–*F*) Uterine inflammatory mRNA expression was significantly greater in LPS-treated mice: *Il-6*, *P* = 0.0005 (*C*); *Il-10*, *P* = 0.0366 (*D*); *Cxcl1*, *P* = 0.0005 (*E*); and *Ccl2*, *P* = 0.0002 (*F*). Simvastatin (40 µg) significantly reduced the expression of these inflammatory mediators (*Il-6,* *P* = 0.0095; *Il-10,* *P* = 0.0328; *Cxcl1,* *P* = 0.0464; and *Ccl2,* *P* = 0.0342), as well as contraction-associated gene *Cx43* (*P* = 0.0143, *G*), compared with LPS alone. *H*) *Cox-2* expression was not altered by LPS or simvastatin; *n* = 6/group, 1-way ANOVA with Dunnett’s *post hoc* test. All data are means ± sem. *P < 0.05, **P < 0.01, ***P < 0.001, ****P < 0.0001.

LPS stimulated a robust elevation of IL-6 in maternal serum (*P* < 0.0001 *vs.* PBS alone) (**Fig. 1*A***), which was significantly attenuated by simvastatin 40 µg (*P* = 0.0213 *vs.* LPS). In contrast, LPS administration did not alter IL-6 levels in the amniotic fluid, and simvastatin treatment had no additional effect (Fig. 1*B*).

We then investigated inflammatory and contraction-associated mRNA expression in the mouse uterus (Fig. 1*C–H*). Unsurprisingly, LPS treatment up-regulated the expression of *Il-6* (*P* = 0.0005), *Il-10* (*P* = 0.0366), *Cxcl1* (*P* = 0.0005), and *Ccl2* (*P* = 0.0002) in the mouse uterus. Treatment with simvastatin 40 µg significantly down-regulated these genes compared with treatment with LPS alone: *Il-6*, *P* = 0.0095; *Il-10*, *P* = 0.0328; *Cxcl1*, *P* = 0.0464; and *Ccl2*, *P* = 0.0342. In addition, the expression of gap junction gene *Cx43* was down-regulated with simvastatin 40 µg treatment (*P* = 0.0143 *vs.* LPS). *Cox-2* mRNA expression was unaltered by LPS and simvastatin treatment. Additional inflammatory genes (*Il-1β*, *Tnf*, and *Cxcl2*) were up-regulated by LPS but unaffected by simvastatin treatment (Supplemental Fig. 2). Protein expression of IL-6 and CX43 in the uterus was unchanged by LPS as well as by simvastatin treatment (Supplemental Fig. 3).

### Simvastatin significantly reduces proinflammatory mediator mRNA and protein expression in human myometrial cells

**Figure 2:**
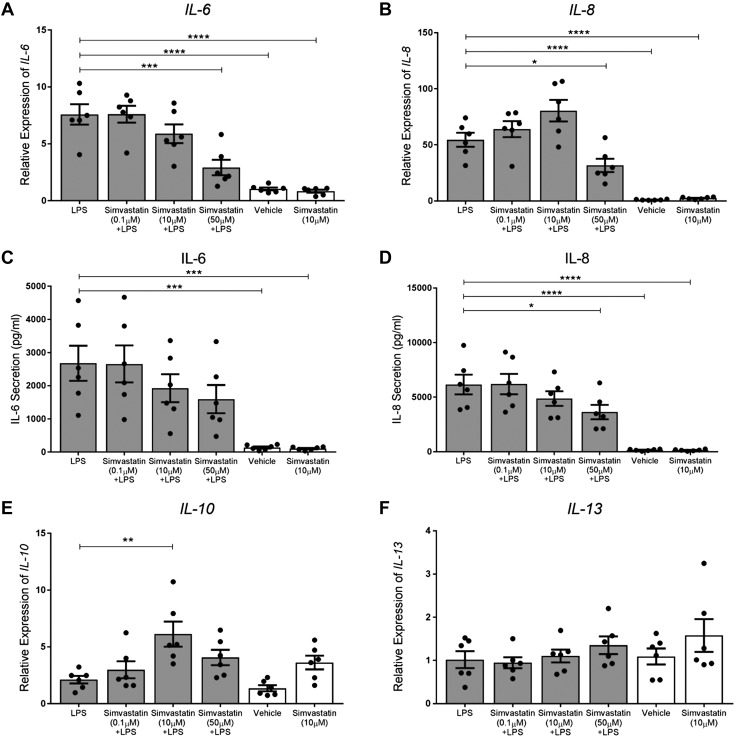
Simvastatin down-regulates proinflammatory mRNA expression in LPS-stimulated human myometrial cells and increases anti-inflammatory mRNA expression. *A*–*D*) LPS robustly up-regulated IL-6 and IL-8 mRNA expression and secretion in human myometrial cells. Post-LPS treatment with 50 µM simvastatin down-regulated *IL-6* (*P* = 0.0003, *A*) and *IL-8* (*P* = 0.041, *B*) expression. *C*, *D*) Post-LPS treatment with 50 µM simvastatin reduced IL-8 (*P* = 0.0427) but not IL-6 concentration. *E*, *F*) Post-LPS treatment with 10 µM simvastatin increased *IL-10* (*P* = 0.0018) but not *IL-13* expression. All data: *n* = 6/group (in duplicate), means ± sem, 1-way ANOVA with Dunnett’s *post hoc* test. *P < 0.05, **P < 0.01, ***P < 0.001, ****P < 0.0001.

We then investigated the effect of simvastatin on inflammation *in vitro* in a human myometrial cell line treated with simvastatin (0.1, 10, 50 µM) either simultaneously with LPS (cotreatment) (Supplemental Fig. 4), 6 h before LPS (pretreatment) (Supplemental Fig. 5), or 6 h after LPS (posttreatment) (**Fig. 2**), for a total of 24 h. Cell metabolic activity, as a measure of cell viability, was unaffected by each of these treatments (Supplemental Fig. 6).

*IL-6* mRNA expression was >6-fold greater in human myometrial cells after LPS stimulation (*P* < 0.0001 *vs.* vehicle). This increase was significantly attenuated by 50 µM simvastatin in the cotreatment, pretreatment, and posttreatment groups (*P* < 0.0001, *P* = 0.0007, and *P* = 0.0003, respectively) (Fig. 2*A* and Supplemental Figs. 4*A* and 5*A*). We found a robust upregulation (>50-fold) of *IL-8* mRNA expression with LPS stimulation in each treatment group (*P* < 0.0001 *vs*. vehicle) (Fig. 2*B* and Supplemental Figs. 4*B* and 5*B*). This up-regulation was significantly reduced with 50 μM simvastatin treatment in both the cotreatment (*P* = 0.0312) and posttreatment (*P* = 0.041) groups compared with LPS alone. We also observed a reduction in IL-6 secretion with 50 µM simvastatin and LPS cotreatment (*P* = 0.0015 *vs.* LPS alone) (Supplemental Fig. 4*C*).

These effects on inflammatory mRNA expression were also observed at the protein level. We found a reduction in IL-6 secretion with 50 μM simvastatin and LPS cotreatment (*P* = 0.0015 *vs*. LPS alone; Supplemental figure 4*C*). However, this reduction was not observed in the pre- and posttreatment groups (Supplemental Fig. 5*C* and Fig. 2*C*). There was a reduction in IL-8 secretion with both cotreatment (*P* = 0.0207) and posttreatment but not pretreatment with 50 μM simvastatin (*P* = 0.0427; Fig. 2*D* and Supplemental Figs. 4*D* and 5*D*).

### Simvastatin significantly increases anti-inflammatory cytokine mRNA expression in human myometrial cells

Interestingly, we observed that 10 µM simvastatin alone was able to up-regulate the expression of the anti-inflammatory genes *IL-10* and *-13* (*P* = 0.0003 and 0.0008 *vs.* LPS, respectively) (Supplemental Fig. 4*E*, *F*). Cotreatment with LPS and both 10 µM simvastatin and 50 µM simvastatin up-regulated *IL-10* and *-13* mRNA expression compared with LPS alone (*P* = 0.0064 and 0.0021). These effects were similar regardless of treatment regimen, although some of the differences were not statistically significant (Fig. 2*E, F* and Supplemental Fig. 5*E*, *F*).

### Simvastatin treatment significantly inhibits both basal and LPS-induced contraction of human myometrial cells

**Figure 3:**
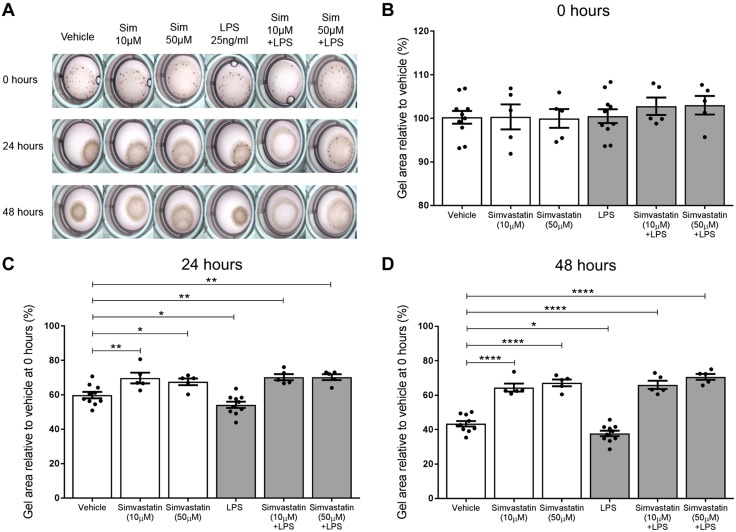
Simvastatin treatment attenuates basal and LPS-induced contraction of myometrial cells embedded in collagen. *A*, *B*) Because these are smooth muscle cells, they established basal contraction, causing the gel size to reduce over time. *C*, *D*) Simvastatin inhibited basal contraction of the myometrial cells, resulting in substantially larger gels compared with the vehicle gels at both 24 h (10 µM, *P* = 0.0059; 50 µM, *P* = 0.027) and 48 h (10 µM, *P* < 0.0001; 50 µM, *P* < 0.0001). Stimulation with LPS induced further contraction, and the gel size was smaller than the vehicle at 24 h (*P* = 0.0293) and 48 h (*P* = 0.0247). When the gels were treated with simvastatin and LPS, contraction was inhibited, and the gel sizes were greater than the vehicle at 24 h (10 µM, *P* = 0.0059; 50 µM, *P* = 0.0059) and 48 h (10 µM, *P* < 0.0001; 50 µM, *P* < 0.0001). PBS and LPS groups, *n* = 10; all other groups, *n* = 5 (4–6 replicates). Mean % relative to vehicle mean at 0 h ± sem, 1-way ANOVA with Holm-Sidak *post hoc* test. *P < 0.05, **P < 0.01, ****P < 0.0001.

Human myometrial cells were embedded in rat tail collagen to assess the effect of simvastatin treatment on the capacity of these cells to contract either alone or within an LPS-stimulated inflammatory environment. Vehicle-treated myometrial cells established a basal contraction, causing the gel to reduce in size. This scenario was evident within 24 h, as the mean gel size reduced to 59.8 ± 1.9% of the baseline gel size (**Fig. 3*A–C***). Simvastatin inhibited the basal contraction of the myometrial cells, resulting in substantially larger gels compared with the vehicle gels. This anticontractile effect was observed with both 10 µM simvastatin (69.7 ± 3.1%, *P* = 0.0059 *vs.* vehicle) and 50 µM simvastatin treatment (67.5 ± 2.0%, *P* = 0.027 *vs.* vehicle). Stimulation with LPS induced further myometrial cell contraction, resulting in a 10.3% smaller gel area than the vehicle at 24 h (54.2 ± 1.8%, *P* = 0.0293 *vs.* vehicle). However, when simvastatin was coadministered with LPS, contraction was inhibited. Gel areas were significantly greater with 10 µM simvastatin (70.2 ± 1.8%, *P* = 0.0059) and 50 µM simvastatin treatment (70.3 ± 1.7%, *P* = 0.0059) compared with the vehicle. After 48 h, the vehicle mean gel size reduced further, and simvastatin treatment continued to inhibit contraction, both alone and in the presence of LPS (Fig. 3*D*).

### Mevalonate and GG-PP but not F-PP supplementation abolish the anticontraction action of simvastatin

**Figure 4:**
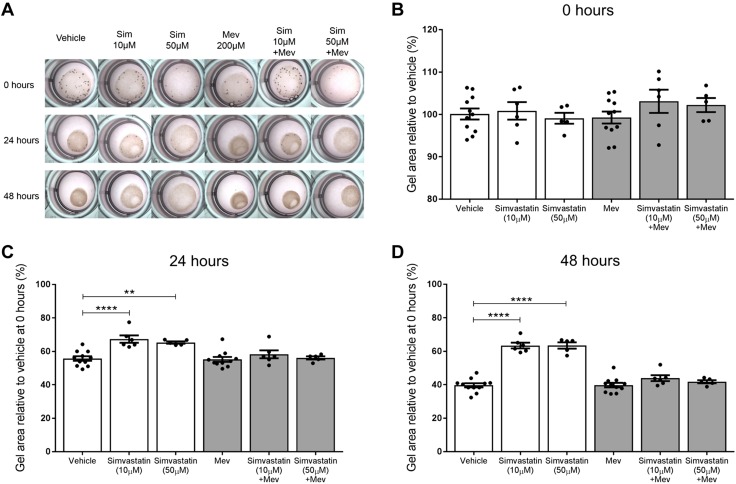
Mevalonate supplementation abolishes the anticontraction effect of simvastatin on myometrial cells. *A*, *B*) Vehicle gels contracted over time, with simvastatin treatment reducing contraction, as evidenced by the significantly larger gel sizes. *C*, *D*) Mevalonate supplementation caused the gels to contract at 24 and 48 h, abolishing the inhibitory effect of the simvastatin. PBS and mevalonate groups, *n* = 10; all other groups, *n* = 5 (4–6 replicates). Mean % relative to vehicle mean at 0 h ± sem, 1-way ANOVA with Dunnett’s *post hoc* test. **P < 0.01, ****P < 0.0001.

**Figure 5:**
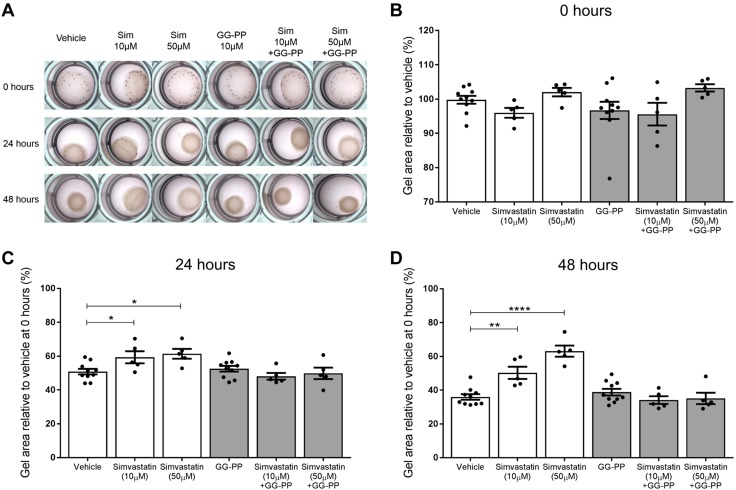
The addition of GG-PP abolishes the anticontraction effect simvastatin has on myometrial cells. When simvastatin-treated cells were supplemented with GG-PP (*A*, *B*), the gels contracted at 24 (*C*) and 48 (*D*) h, thus abolishing the anticontractile effect of simvastatin. PBS and GG-PP groups, *n* = 10; all other groups, *n* = 5 (4–6 replicates). Mean % relative to vehicle mean at 0 h ± sem, 1-way ANOVA with Dunnett’s *post hoc* test. *P < 0.05, **P < 0.01, ****P < 0.0001.

**Figure 6:**
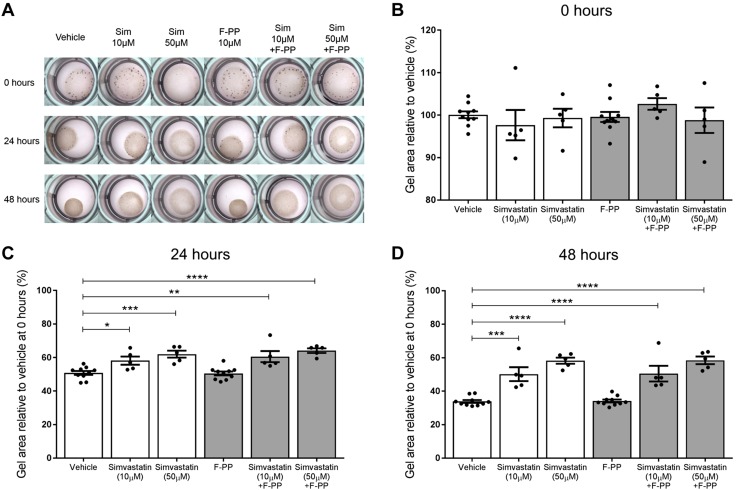
F-PP supplementation does not abolish the anticontraction effect of simvastatin on myometrial cells. When myometrial cells were treated with 10 or 50 µM simvastatin and supplemented with F-PP (*A*, *B*), the gel sizes remained significantly larger than the vehicle gels at 24 h (10 µM, *P* = 0.0024; 50 µM, *P* < 0.0001) (*C*) and 48 h (10 µM, *P* = 0.0001; 50 µM, *P* < 0.0001) (*D*). PBS and F-PP groups, *n* = 10; all other groups, *n* = 5 (4–6 replicates). Mean % relative to vehicle mean at 0 h ± sem, 1-way ANOVA with Dunnett’s *post hoc* test. *P < 0.05, **P < 0.01, ***P < 0.001, ****P < 0.0001.

To investigate a mechanism by which simvastatin may be exerting its anticontraction effect, we supplemented the collagen gels with metabolites from the mevalonate pathway, which is the metabolic pathway that statins inhibit. When mevalonate was added with either 10 or 50 µM simvastatin, the gels contracted, abolishing the inhibitory effect of simvastatin on contractions. This effect was observed at both 24 and 48 h, with no significant difference in gel size compared with the vehicle (**Fig. 4**). Similarly, when simvastatin-treated gels were supplemented with GG-PP, the gels contracted, again demonstrating abolition of the anticontraction effect of simvastatin (**Fig. 5**). In contrast, F-PP supplementation did not inhibit the anticontraction effect of simvastatin (**Fig. 6**). The gel sizes were still significantly larger than the vehicle gels at 24 h with 10 µM simvastatin (*P* = 0.0024) and 50 µM simvastatin (*P* < 0.0001). This effect was also observed at 48 h with 10 µM simvastatin (*P* = 0.0001) and 50 µM simvastatin (*P* < 0.0001).

### Simvastatin reduces MLC phosphorylation *via* inhibition of the Rho/Rho-associated protein kinase pathway

**Figure 7:**
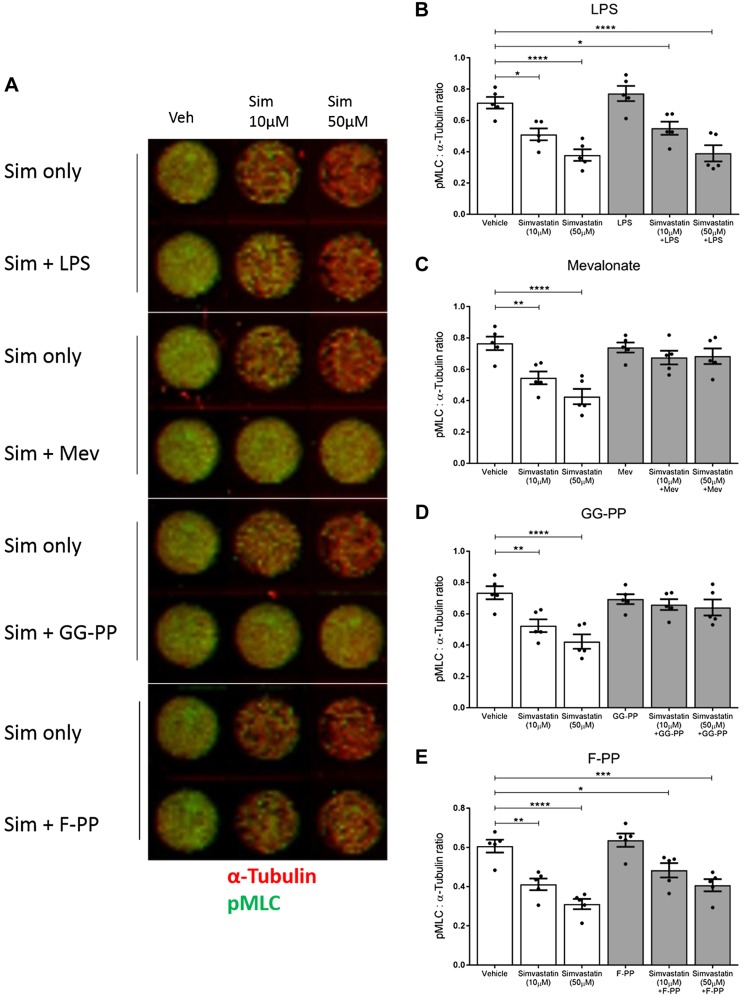
Simvastatin reduces pMLC concentration, which is rescued by mevalonate and GG-PP but not F-PP supplementation. *A*, *B*) Simvastatin treatment reduced the phosphorylation of MLC both alone (10 µM, *P* = 0.0112; 50 µM, *P* < 0.0001) and in the presence of LPS (10 µM, *P* = 0.0493; 50 µM, *P* < 0.0001). LPS itself did not affect the phosphorylation of MLC. *A*, *C*, *D*) Mevalonate and GG-PP supplementation abolished the effect of simvastatin, and the pMLC concentrations were similar to the vehicle-treated cells. *A*, *E*) F-PP supplementation did not alter the action of simvastatin, and pMLC concentration remained significantly reduced compared with the vehicle (10 µM, *P* = 0.0452; 50 µM, *P* = 0.0008). All data: *n* = 5/group (in triplicate); means ± sem, 1-way ANOVA with Dunnett’s *post hoc* test. *P < 0.05, **P < 0.01, ***P < 0.001, ****P < 0.0001.

We used the in-cell Western technique to investigate the impact of simvastatin on phosphorylation of MLC. Simvastatin treatment reduced pMLC concentration both alone [10 µM simvastatin (*P* = 0.0112) and 50 µM simvastatin (*P* < 0.0001) *vs.* vehicle] and in the presence of LPS [10 µM simvastatin (*P* = 0.0493) and 50 µM simvastatin (*P* < 0.0001) *vs.* vehicle]. LPS alone did not affect the expression of pMLC (**Fig. 7*A, B***). Once again, when cells were treated with simvastatin and received either mevalonate or GG-PP supplementation, the effect of simvastatin was abolished, and the pMLC levels were akin to the vehicle-treated cells (Fig. 7*A*, *C*, *D*). When cells were treated with simvastatin and supplemented with F-PP, pMLC remained significantly reduced compared with the vehicle (10 µM simvastatin, *P* = 0.0452; 50 µM simvastatin, *P* = 0.0008). Thus, the addition of F-PP did not prevent simvastatin from inhibiting the phosphorylation of MLC (Fig. 7*A, E*), complementing the results of the collagen gel contraction assay.

## DISCUSSION

We present comprehensive evidence from preclinical models that statins (*e.g.*, simvastatin) should be considered as therapeutic agents for the treatment of PTL in women. Specifically, we have shown that treatment with simvastatin reduces the incidence of early delivery in a robust and reproducible mouse model of PTB. We speculate that the ability of simvastatin to reduce systemic and uterine inflammation in this model may explain the lower incidence of PTB. These findings appear to agree with preliminary findings reported by Gonzalez *et al.* (16) whereby 20 µg simvastatin was found to prevent PTB in a mouse model of PTB induced by intravaginal administration of LPS (serotype 055:B5). Unfortunately, neither we nor any other group has been able to replicate PTB through vaginal administration of LPS (24). Our study not only used a validated model of PTB in a larger cohort of mice, but we report on the survival of the pups immediately after delivery, identify an inhibitory effect of simvastatin on maternal inflammation, and defined the mechanism. These studies pave the way for definitive human studies of the effects of statins on PTL.

Interestingly, simvastatin did not have a dose-dependent effect on time to delivery or the percentage of live-born pups. However, the higher dose of 40 μg simvastatin was more effective than the 20-μg treatment at reducing inflammation and contraction-associated genes. We showed that 40 µg simvastatin treatment lowered the maternal serum concentration of IL-6 induced by LPS stimulation, as well as reduced the mRNA concentration of cytokines and chemokines in the uterus. However, IL-6 protein in the uterus was unaltered by LPS, as well as simvastatin, when assessed according to Western blot. This outcome could have been due to the timing of the tissue collection, as the majority of IL-6 may have been released into the bloodstream or surrounding tissues. In amniotic fluid, IL-6 concentration was also unchanged by LPS, as well as simvastatin. This finding may be a limitation of our PTB mouse model, as IL-6 levels are commonly found to be elevated in cases of PTL in women. However, we only performed collections 6 h after LPS administration; it is thus unknown if inflammatory markers may have been increased at a later time point.

The anti-inflammatory results reported here are broadly consistent with other animal models in which statins have reduced inflammation and altered the immune response (30–33). More specifically, our observations are in agreement with a study in which pretreatment with simvastatin reduced inflammation in a mouse model of PTB induced by the administration of LPS into the peritoneum (34). The effect of this treatment on the incidence of PTB was not reported, and there have been no follow-up studies, suggesting that pretreatment with simvastatin did not prevent PTB in this model. In the present study, mice received both a pretreatment and post-LPS treatment with simvastatin. This treatment protocol was chosen due to its previously reported success in other mouse models of pregnancy disorders (16, 21, 35). It is unknown whether treatment at either time point alone would also have been successful. Simvastatin and its active metabolite both have a short half-life of ∼2–4 h; thus, it could be hypothesized that the post-LPS treatment may have been the more influential treatment for the prevention of PTB and to lower inflammation (36, 37). Future experiments addressing this topic will be important for determining how statins could best be applied clinically. In addition, an alternative route of treatment should also be investigated, as statins would normally be administered orally in women. In subsequent mouse studies, oral treatments can be given *via* gavage.

Our observations *in vitro* further support the use of simvastatin as an anti-inflammatory treatment. We performed cotreatments, pretreatments, and posttreatments to better understand the potential clinical application of statin treatment, and whether it should be used as a preventative measure for high-risk patients or given at the onset of PTL. Simvastatin reduced proinflammatory mediator gene expression and secretion, as well as increased the expression of anti-inflammatory genes. These effects were evident with both pretreatment and posttreatment, suggesting that statins could be effective for both the prevention and treatment of PTL. These anti-inflammatory effects have been well documented in other cell types, such as vascular smooth muscle cells, macrophages, epithelial cells, and endometriotic cells (38–42). Simvastatin treatment reduced the secretion of proinflammatory cytokines from human fetal membranes stimulated with LPS, with simvastatin pretreatment being the most effective regimen compared with cotreatment and posttreatment (43). To our knowledge, our study provides the first evidence that simvastatin has anti-inflammatory effects on human myometrial cells. Although the mechanisms responsible for these anti-inflammatory effects have not been fully elucidated, it is likely that they are a result of the inhibition of protein isoprenylation within the mevalonate pathway (44). Other studies have shown that statins can interfere with the nuclear translocation and binding activity of NF-κB (45, 46).

It is well established that there is an increase in proinflammatory mediators, such as chemokines, cytokines, and prostaglandins, as well as contraction-associated proteins, in the uterus during labor (47–49), and an association between inflammation and the stimulation of myometrial contractions has emerged in recent years (27, 50). In our mouse model, we found that simvastatin reduced the expression of *Cx43*, a gap junction gene, in the uterus. The impact of statins on *Cx43* mRNA expression has not been addressed in other animal models of PTB. We observed no differences in CX43 at the protein level, but it may be that the translocation of connexins to the cell surface was affected by LPS and simvastatin treatment. In addition, neither LPS nor simvastatin altered *Cox-2* mRNA expression at this time point. The effect of these treatments on prostaglandin production could be investigated in future studies. More specifically, we showed that simvastatin attenuated the contraction of human myometrial smooth muscle cells alone and within an inflammatory environment stimulated by LPS. The anticontraction effect of statins has been reported in other cell types and tissues, such as vascular smooth muscle cells, aortic rings, and human endometriotic stromal cells (15, 17–19, 51, 52). Furthermore, pretreatment with simvastatin reduced the frequency of spontaneous and C5a-induced contraction of human myometrial strips, as well as in myometrial tissue collected from simvastatin-treated mice, but the effect on the intensity of the contractions was not investigated (16). Notably, the effect of statins on LPS-induced contraction has not previously been investigated. Current tocolytic drugs used to inhibit myometrial contractions are generally ineffective at substantially delaying labor (11, 12). We have shown that simvastatin is capable of inhibiting contractions, even when in the presence of inflammation; this finding provides further evidence that simvastatin may be a beneficial therapeutic agent to maintain or restore uterine quiescence, a key quality for a PTL therapeutic.

**Figure 8:**
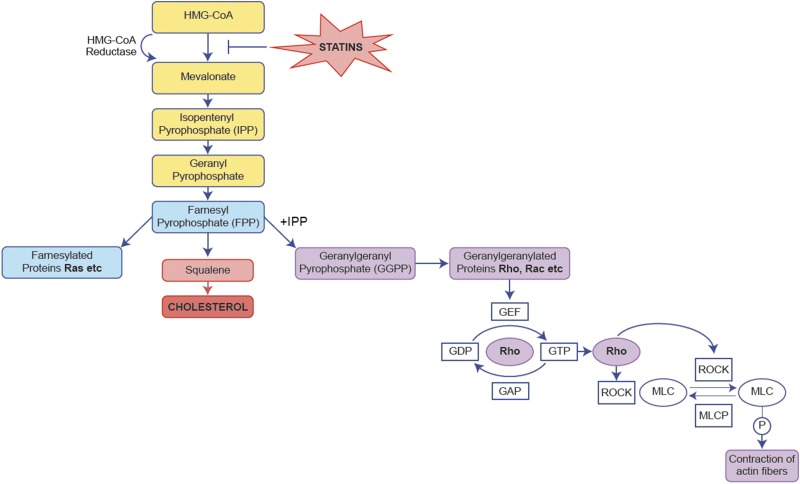
Simvastatin reduces myometrial cell contraction by inhibiting the Rho/ROCK pathway *via* the attenuation of GG-PP production. Mevalonate and GG-PP but not F-PP supplementation abolished the anticontraction effects of simvastatin. By attenuating the production of GG-PP, simvastatin inhibited the geranylgeranylation of Rho. Thus, simvastatin indirectly prevented the phosphorylation of MLC by ROCK, subsequently inhibiting actin fiber contraction. HMG-CoA, 3-hydroxy-3-methylglutaryl-coenzyme A; MLCP, MLC phosphatase; ROCK, RhoA kinase.

Further demonstrating that simvastatin can inhibit the contraction of myometrial cells, we also propose a mechanism for this effect (**Fig. 8**). Supplementation with metabolites of the mevalonate pathway allowed us to pinpoint the branch of the pathway targeted by simvastatin. Mevalonate supplementation abolished the anticontraction effect of simvastatin, confirming that simvastatin is in fact targeting the mevalonate pathway. Mevalonate is converted to sterol and nonsterol isoprenoids, of which F-PP and GG-PP are components. The isoprenylation of GG-PP is crucial for the activation of small GTPases, such as Rho, Rac, and Cdc42, which are associated with cellular functions, including gene expression and remodeling of the actin cytoskeleton (44, 53–55). GG-PP abolished the anticontraction actions of simvastatin, suggesting that simvastatin was having its effect by inhibiting the geranylgeranylation of small GTPases. In contrast, F-PP supplementation did not reverse the effects of simvastatin, implying these effects were not the result of inhibiting cholesterol or farnesylated protein production. Although F-PP is the precursor to GG-PP, both F-PP and isopentenyl pyrophosphate (I-PP) must be present to produce GG-PP (53). When F-PP alone is added back into a statin treatment experiment, I-PP is not present; thus, GG-PP cannot be produced in this artificial setting. This scenario therefore allows the differential effects of these intermediates to be identified *in vitro*. It can be hypothesized that by supplementing simvastatin experiments with both F-PP and I-PP, GG-PP may be produced, and the actions of simvastatin could be reversed. The results of these mechanism studies confirm and extend those in the literature (19, 56, 57). This mechanism has also been observed with other statins, in which the contraction of rat myofibers and cytoskeletal rearrangement were affected due to the inhibition of geranylgeranylation and not farnesylation (58, 59).

The Rho/Rho-associated protein kinase (ROCK) pathway has long been associated with smooth muscle contraction, and the importance of ROCK-mediated MLC phosphorylation for myometrial contractility has been previously reported (60, 61). Active Rho binds to ROCK after geranylgeranylation, the conversion of GDP to GTP, and membrane anchoring (62). Once ROCK is activated, it can interfere with MLC kinase and MLC phosphatase, or ROCK can phosphorylate MLC directly at the Ser-19 site (63), facilitating actin–myosin contraction (64–66). Simvastatin treatment reduced the phosphorylation of MLC, validating its anticontractile effect at a molecular level. However, LPS did not alter pMLC levels at the time point we investigated, despite showing that LPS significantly reduces the collagen gel size in the contraction assay at 24 and 48 h, although we have previously published details regarding the ability of another serotype of LPS to modestly increase levels of pMLC at 48 h (27). Again, mevalonate and GG-PP, but not F-PP, abolished the effects of simvastatin. We therefore propose that simvastatin attenuated myometrial cell contraction by inhibiting the isoprenylation of Rho by GG-PP, preventing the activation of ROCK, which subsequently prevented the phosphorylation of MLC (Fig. 8). Other studies have identified the inhibition of the Rho/ROCK pathway and the subsequent prevention of MLC phosphorylation as the mechanism responsible for contraction inhibition by statins, adding strength to our findings (51, 57, 67–70). Statins can also affect intracellular Ca^2+^ concentration, which may provide another mechanism for these effects (52, 71, 72).

We and others are now planning clinical trials of statins to prevent PTB in human pregnancy. Although concerns regarding statin use during pregnancy were raised after a retrospective observational study by Edison and Muenke, which suggested that simvastatin use during the first trimester was associated with fetal limb defects, recent, larger studies have challenged and criticized these findings (73–76). In addition, a number of systematic reviews have also failed to find evidence of teratogenicity (77–79). However, treatment of PTL is likely to occur in the second or third trimester when organogenesis is mostly complete. Furthermore, it can be argued that the benefits may outweigh the risks, as PTB itself is associated with neonatal mortality and lifelong morbidity. Small, human trials have investigated the effect of pravastatin on women at high risk of preeclampsia, and no safety concerns have been reported (20, 23). However, there is uncertainty about whether the safety profiles of all statins are similar, as the pharmacokinetic profiles of individual statins vary (36).

It is conceivable that simvastatin may also protect against inflammation-induced fetal injury, as statins reportedly prevented fetal death in mouse models of recurrent miscarriage and antiphospholipid syndrome, as well as preventing abnormalities in the cortex of the fetal brain in a mouse model of PTB (21, 22, 35). Protecting against fetal injury could prevent the development of common, long-term behavioral and neurologic disorders associated with prematurity such as cerebral palsy. Although the number of pups born alive did increase with simvastatin treatment in this study, the percent increase compared with the LPS group did not reach significance. However, these experiments were powered to show statistical significance in the time to delivery between the LPS and simvastatin treatment groups. Therefore, further research should investigate the effect of simvastatin on neonatal outcome, particularly any neurodevelopmental effects.

In summary, the present study provides the rationale for further evaluation of the efficacy of simvastatin as a novel treatment for PTL. We have shown that simvastatin treatment reduced the incidence of PTB in an intrauterine, LPS-induced mouse model, as well as reduced inflammation in the uterus of these mice. Simvastatin also reduced LPS-induced inflammation in human myometrial cells and inhibited the contraction of these cells, both basally and within an inflammatory environment, *via* the inhibition of the Rho/ROCK pathway. Simvastatin treatment exhibited a number of useful properties, suggesting this drug would be an ideal candidate for the treatment of PTL by targeting underlying inflammation, inhibiting myometrial contractions, and subsequently preventing PTB.

## Supplementary Material

This article includes supplemental data. Please visit *http://www.fasebj.org* to obtain this information.

## Abbreviations

Cx: connexin
F-PP: farnesyl pyrophosphate
GG-PP: geranylgeranyl pyrophosphate
I-PP: isopentenyl pyrophosphate
MLC: myosin light chain
MTT: 3-(4,5-dimethylthiazol-2-*yl*)-2,5-diphenyltetrazolium bromide
pMLC: phosphorylated myosin light chain
PTB: preterm birth
PTL: preterm labor
qRT-PCR: quantitative RT-PCR
ROCK: Rho-associated protein kinase
