# Supplementary material for: Repurposing simvastatin as a therapy for preterm labor: evidence from preclinical models
Source: FASEB J. 2018 Oct 12;33(2):2743–58. doi: 10.1096/fj.201801104R (PMC6338657; doi:10.1096/fj.201801104R)
Supplement: Supplementary file 1 [file fj.201801104R.sf1.docx]

**
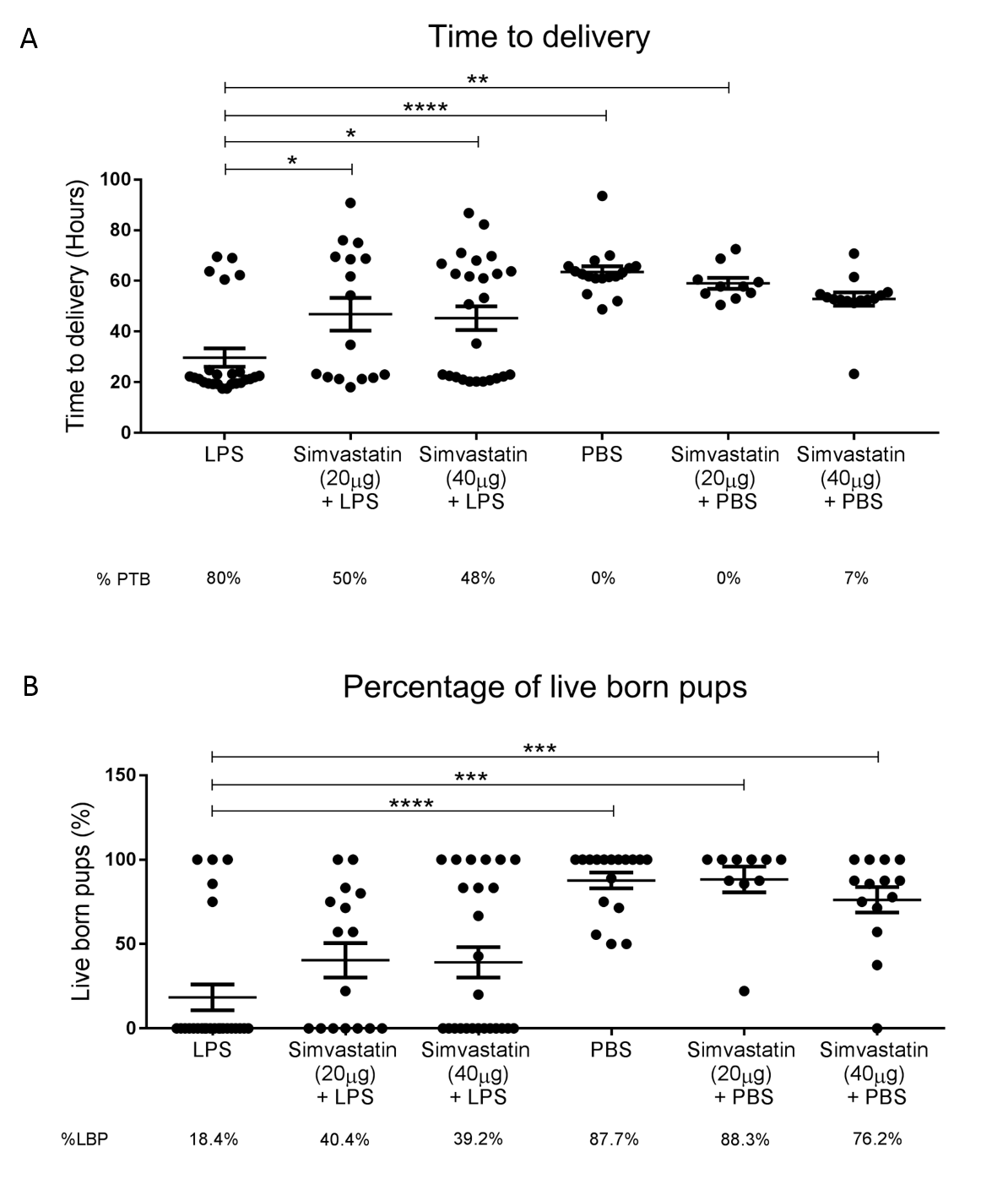
**

**Supplemental figure 1. Simvastatin reduces the incidence of early delivery in a mouse model of PTB.** (A) Mice receiving intrauterine LPS delivered significantly earlier than PBS treated mice (p<0.0001). Simvastatin treatment significantly prolonged pregnancy following LPS administration (20µg p=0.0383 and 40µg p=0.0469). n=10-25 dams/group, Kruskal-Wallis with Dunn's post hoc test. (B) There was a significant reduction in the percentage of pups born alive following LPS treatment, compared to PBS and simvastatin control mice (p<0.0001). n=10-25 dams/group, one-way ANOVA with Dunnett’s post hoc test. All data mean ± SEM, *p<0.05 **p<0.01, ***p<0.001, ****p<0.0001.

**
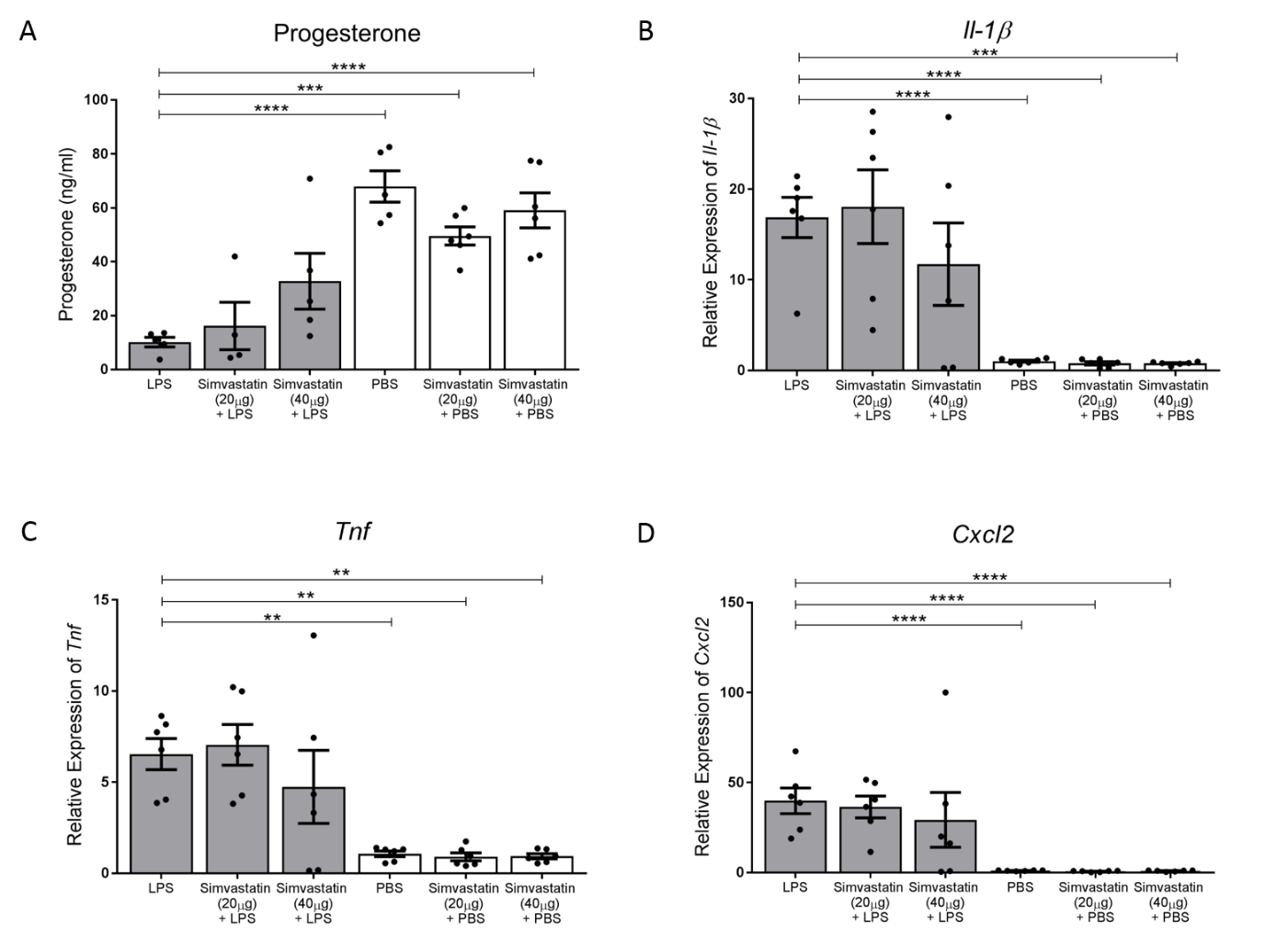
**

**Supplemental figure 2. Serum progesterone concentration and additional mRNA analyses of the uterus in a mouse model of PTB.** (A) Simvastatin alone did not affect circulating progesterone concentration. LPS caused progesterone withdrawal (p<0.0001 vs PBS). n=4-6/group, mean ± SEM, **p<0.01, p<0.001 ****p<0.0001, one-way ANOVA with Dunnett’s post hoc test. (B-D) LPS upregulated *Il-1β*, *Tnf* and *Cxcl2* mRNA expression in the mouse uterus. Simvastatin treatment did not significantly alter the expression of these genes. n=6, mean ± SEM, *p<0.05, **p<0.01, ***p<0.001 ****p<0.0001, one-way ANOVA with Dunnett’s post hoc test.


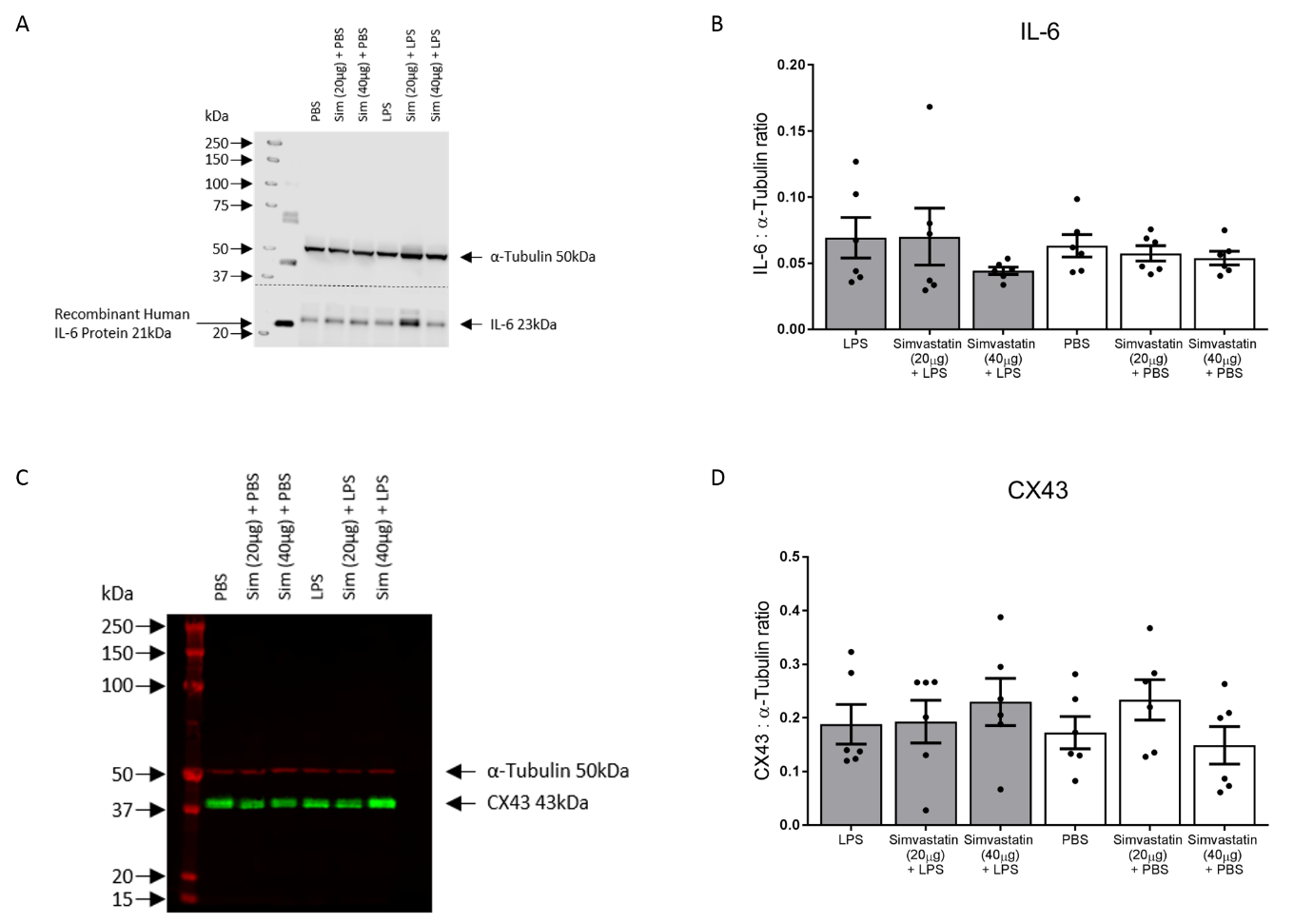


**Supplemental figure 3. IL-6 and CX43 protein abundance are unchanged in the mouse uterus 6 hours after intrauterine injection.** (A) Representative chemiluminescent image for IL-6. Recombinant human IL-6 protein was included as a positive control. Representative image was stitched together (broken line), as α-Tubulin and IL-6 protein required different ECL incubation times (30 seconds and 5 minutes, respectively). (B) IL-6 concentration was not altered by LPS or simvastatin treatment in the uterus 6 hours after intrauterine PBS/LPS administration. (C) Representative fluorescent image for CX43. (D) Protein levels of CX43 were unaltered by either LPS or simvastatin 6 hours after intrauterine treatment with LPS/PBS. n=6, mean ± SEM.

**
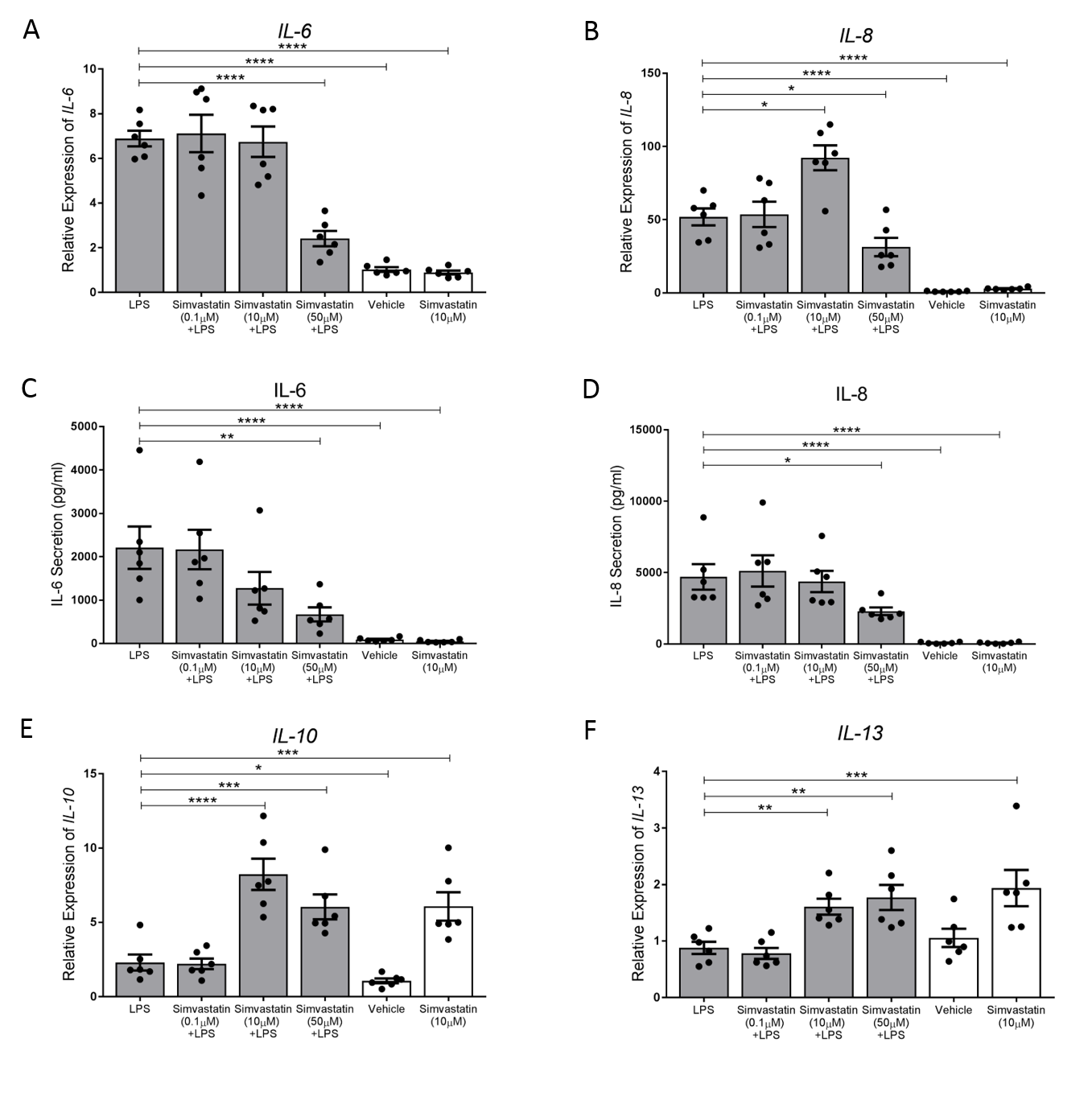
**

**Supplemental figure 4. Co-treatment with LPS and simvastatin alters inflammatory mRNA expression and secretion in human myometrial cells.** (A, B) Co-treatment with LPS and 50µM simvastatin downregulated *IL-6* (p<0.0001) and *IL-8* (p=0.0312) compared to LPS alone. (C, D) Co-treatment with LPS and 50µM simvastatin reduced IL-6 (p=0.0015) and IL-8 secretion (p=0.0207) compared to LPS. (E) Simvastatin alone upregulated *IL-10* mRNA expression (p=0.0003 vs LPS). Co-treatment with LPS and both 10µM simvastatin and 50µM simvastatin also upregulated *IL-10* expression (p<0.0001 and p=0.0003). (F) Simvastatin alone (p=0.0008) and co-treatment with LPS and 10µM simvastatin (p=0.0064) and 50µM simvastatin (p=0.0021) upregulated *IL-13* expression. All data n=6/group (in duplicate), mean ± SEM, *p<0.05 **p<0.01, ***p<0.001, ****p<0.0001, one-way ANOVA with Dunnett’s post hoc test.


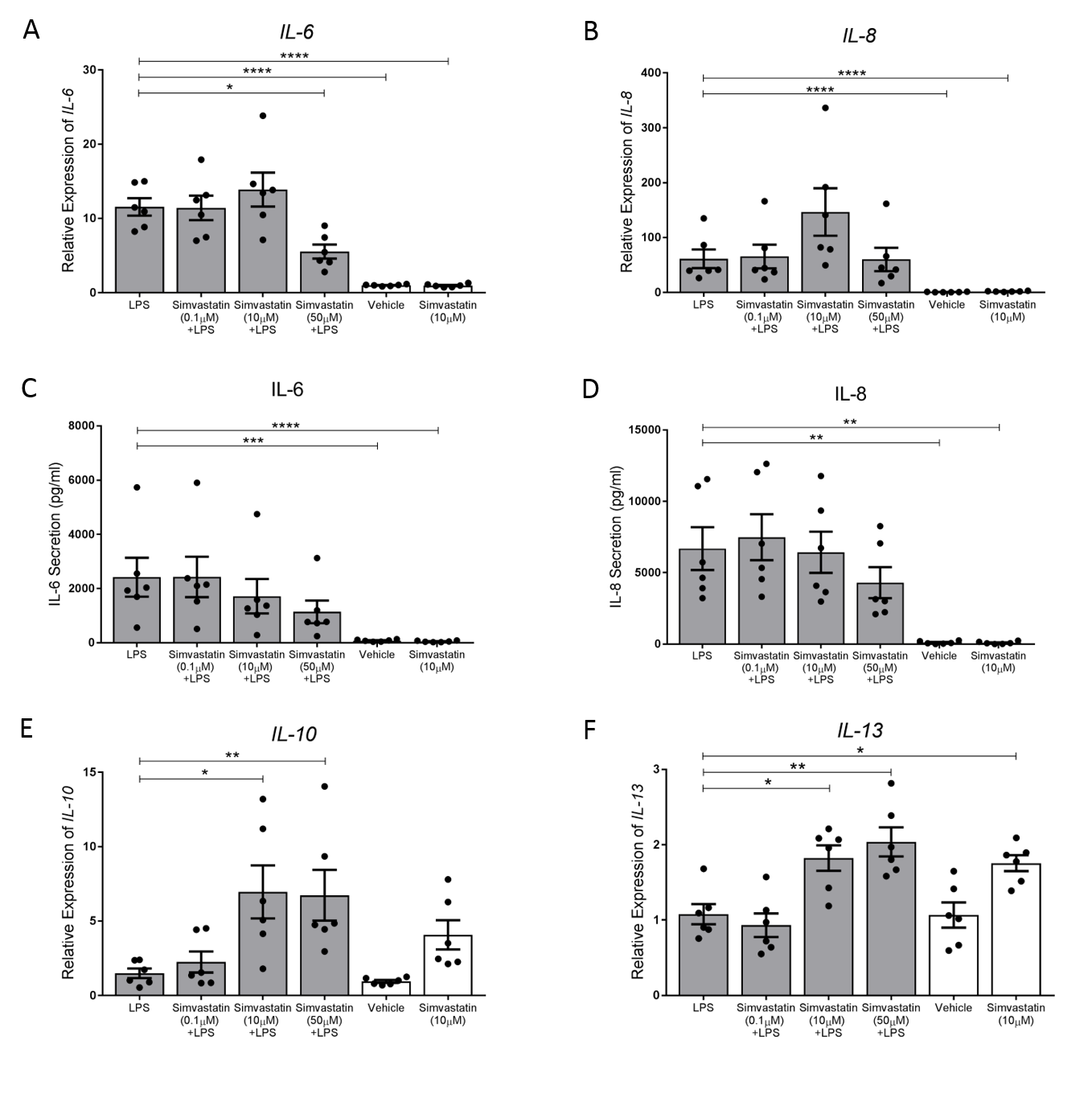


**Supplemental figure 5. Pre-treatment with simvastatin alters LPS-induced** **inflammatory mRNA expression and secretion in human myometrial cells.** (A, B) Pre-treatment with 50µM simvastatin downregulated *IL-6* (p=0.0007) but not *IL-8* expression. (C, D) Pre-treatment with simvastatin did not affect IL-6 or IL-8 secretion at 24 hours. (E) Expression of *IL-10* was increased by pre-treatment with 10µM simvastatin (p=0.0165) and 50µM simvastatin (p=0.0027). (F) Simvastatin alone upregulated *IL-13* expression (p=0.0235), as did pre-treatment with 10µM simvastatin (p=0.0064) and 50µM simvastatin (p=0.0021). All data n=6/group (in duplicate), mean ± SEM, *p<0.05 **p<0.01, ***p<0.001, ****p<0.0001, one-way ANOVA with Dunnett’s post hoc test.

**
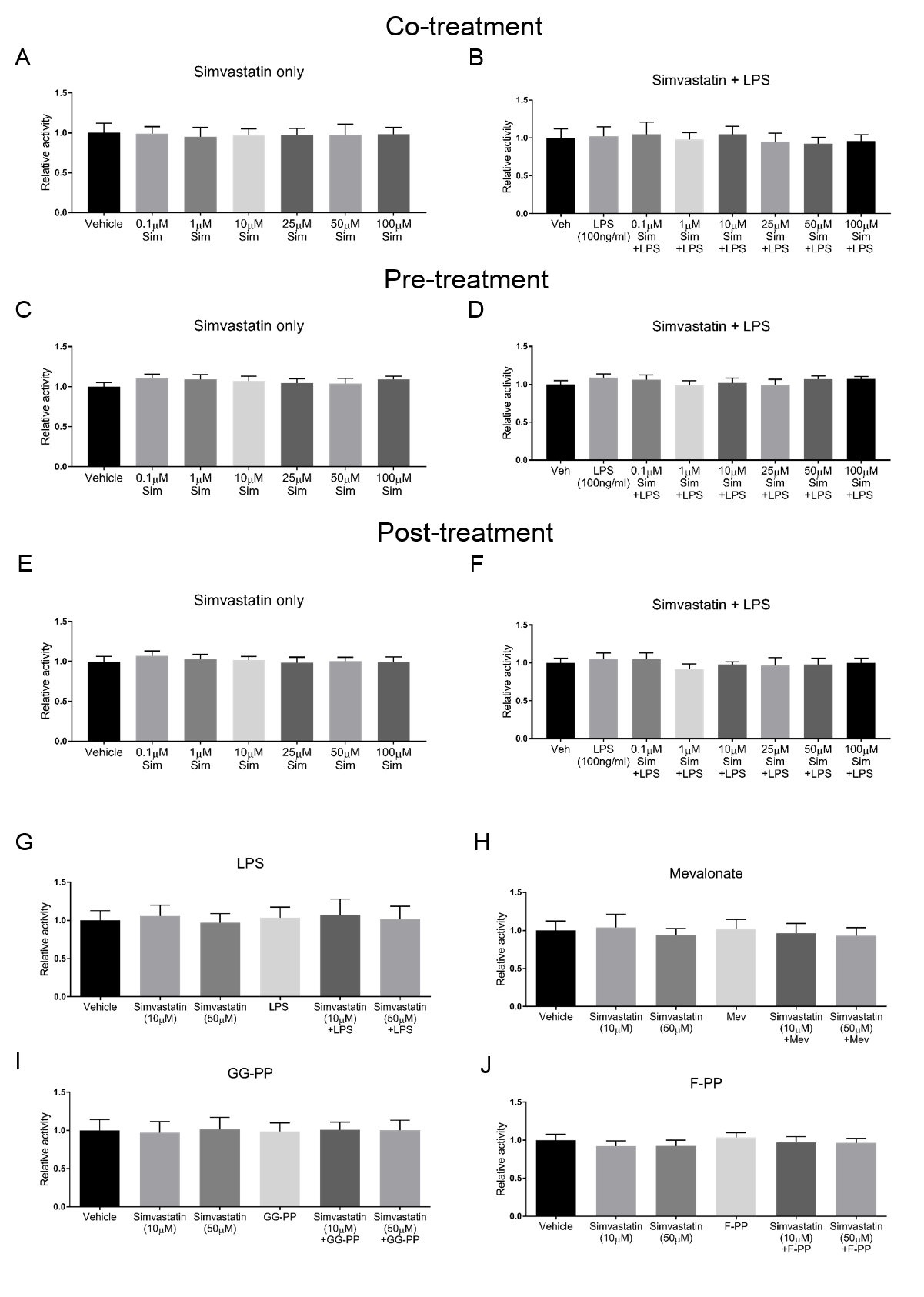
Supplemental figure 6. Myometrial cell metabolic activity.** (A-F) Human myometrial cell viability was unaffected by 24 hours of LPS and simvastatin co-, pre- and post-treatment. n=6 (in triplicate), mean ± SEM. (G-J) Myometrial cell viability was not impacted by treatment with simvastatin and LPS, mevalonate, GG-PP or F-PP over 48 hours. n=4 (in triplicate) mean ± SEM.
